# Modeling vanishing white matter disease with patient‐derived induced pluripotent stem cells reveals astrocytic dysfunction

**DOI:** 10.1111/cns.13107

**Published:** 2019-02-05

**Authors:** Ling Zhou, Peng Li, Na Chen, Li‐Fang Dai, Kai Gao, Yi‐Nan Liu, Li Shen, Jing‐Min Wang, Yu‐Wu Jiang, Ye Wu

**Affiliations:** ^1^ Department of Pediatrics Peking University First Hospital Beijing China; ^2^ Department of Cell Biology, School of Basic Medical Sciences, Stem Cell Research Center Peking University Beijing China

**Keywords:** astrocytes, induced pluripotent stem cells, neural stem cells, neurons, oligodendrocytes, vanishing white matter disease

## Abstract

**Aims:**

Vanishing white matter disease (VWM) is an inherited leukoencephalopathy in children attributed to mutations in *EIF2B1–5, *encoding five subunits of eukaryotic translation initiation factor 2B (eIF2B). Although the defects are in the housekeeping genes, glial cells are selectively involved in VWM. Several studies have suggested that astrocytes are central in the pathogenesis of VWM. However, the exact pathomechanism remains unknown, and no model for VWM induced pluripotent stem cells (iPSCs) has been established.

**Methods:**

Fibroblasts from two VWM children were reprogrammed into iPSCs by using a virus‐free nonintegrating episomal vector system. Control and VWM iPSCs were sequentially differentiated into neural stem cells (NSCs) and then into neural cells, including neurons, oligodendrocytes (OLs), and astrocytes.

**Results:**

Vanishing white matter disease iPSC‐derived NSCs can normally differentiate into neurons, oligodendrocytes precursor cells (OPCs), and oligodendrocytes in vitro. By contrast, VWM astrocytes were dysmorphic and characterized by shorter processes. Moreover, δ‐GFAP and αB‐Crystalline were significantly increased in addition to increased early and total apoptosis.

**Conclusion:**

The results provided further evidence supporting the central role of astrocytic dysfunction. The establishment of VWM‐specific iPSC models provides a platform for exploring the pathogenesis of VWM and future drug screening.

## 1 INTRODUCTION

Human induced pluripotent stem cells (iPSCs) have the potential for self‐renewal and multilineage differentiation.^1^ iPSCs have been a useful tool for establishing disease‐specific models for exploring the mechanisms of specific diseases by using the patients’ own cells, such as dermal fibroblasts and urothelia.

Vanishing white matter disease (VWM) is one of the most prevalent inherited leukoencephalopathies in childhood and is characterized by progressive motor deterioration with episodic aggravation predisposed by stress, such as febrile infection, minor head trauma, and acute fright.^2^, ^3^, ^4^, ^5^ The disease‐causing genes are *EIF2B1–5*, encoding the subunits of eukaryotic translation initiation factor 2B (eIF2B α, β, γ, δ, and ε), which are responsible for the initiation of eukaryotic protein translation.^6^, ^7^, ^8^, ^9^ Although the defects are in the housekeeping genes, glial cells are selectively affected in VWM. The definite mechanism of VWM remains unrevealed, and no iPSC model has been established in VWM. In this study, fibroblasts from two VWM children were reprogrammed into iPSCs for the first time by using virus‐free nonintegrating episomal vector system.^10^, ^11^, ^12^ Control and VWM iPSCs were sequentially differentiated into neural stem cells (NSCs) and then into neurons, oligodendrocytes, and astrocytes, respectively. We tried to determine whether there is a disturbance in the differentiation processes of VWM NSCs into different lineages.

## 2 MATERIALS AND METHODS

### 2.1 Isolation of VWM patients’ dermal fibroblasts and cell culture

Human dermal fibroblasts (HDFs) from the dermis of two VWM children were used for the establishment of the VWM1‐iPSCs and VWM2‐iPSCs. Two age‐matched control iPSCs (C1 and C2) were obtained from the Stem Cell Research Center at Peking University. The HDFs were cultured in a standard medium containing high‐glucose DMEM supplemented with 10% fetal bovine serum (FBS). iPSCs were maintained in Essential 8 medium (E8, Life Technology, Carlsbad, CA, USA) on Matrigel (BD Biosciences, San Jose, CA, USA). iPSCs were passaged every 3‐5 days by EDTA (Life Technology).

### 2.2 Generation of VWM iPSCs from HDFs

Yamanaka episomal plasmids used in experiments, including pCXLE‐hOCT3/4‐SHP53 (#27‐007), PCXLE‐hSK (#27078), pCXLE‐hUL (#27080), and pCXLE‐EGFAP (#27082) (encoding OCT4, SOX2, Lin28, L‐MYC, and KLF4). HDFs (5 × 10^5^) were counted and resuspended in the Nucleofector solution supplied in the Amaxa Nucleofector kit (Lonza, Basel, Switzerland). The episomal plasmids were added to the cell suspensions at 10 μg each per reaction and were cotransfected using the program U‐023 on the Amaxa Nucleofector device. The cells were then transferred to a Matrigel‐coated 60‐mm culture dish and cultured in the fibroblast medium for 3 days, and then changed to N2B27 medium supplemented with bFGF (Invitrogen, Carlsbad, CA, USA), Y‐27632 (Sigma, St. Louis, MO, USA), CHIR99021 (Selleck, Houston, TX, USA), PD0325901 (Selleck), hLIF (Invitrogen), and A‐83‐01 (Selleck). On day 12, the medium was changed to E8. By 20‐30 days post‐transfection, clones were picked and cultured in E8 medium.

### 2.3 Characterization of the iPSCs

Chromosomal G‐band analysis was performed on VWM iPSCs with more than 10 passages. Alkaline phosphatase (ALP) staining was performed by using an ALP kit (Invitrogen). Teratoma assay was performed to test the in vivo differentiation potential of iPSCs. The undifferentiated iPSCs (1 × 10^7^ cells/mL) were suspended in PBS and injected subcutaneously into the posterior limbs of 4‐week‐old NOD/SCID mice. Two months after injection, the teratomas were dissected and fixed in 4% paraformaldehyde. The paraffin‐embedded tissue was sliced and stained with hematoxylin and eosin.

### 2.4 RNA isolation and quantitative real‐time PCR

Total RNA was extracted using Trizol reagent (Invitrogen). cDNA was synthesized using a reverse transcription kit (Promega, Madison, WI, USA). Quantitative PCR was performed using SYBR Green Real‐time PCR Master Mix (Promega). The relative expression levels were normalized to those of GAPDH based on the delta‐Ct method. The forward and reverse primers for real‐time PCR are as follows: GAPDH (CTCTCTGCTCCTCCTGTTCGAC, TGAGCGATGTGGCTCGGCT); Total GFAP(AGAAGCTCCAGGATGAAACC, TTCATCTGCTTCCTGTCTATAGG); α‐GFAP (AGAGGTCATTAAGGAGTCCA, CAACTATCCTGCTTCTGCTC); δ‐GFAP(CCTACAGGAAGCTGCTAGAG, GCGTTCCATTTACAATCTGGT).

### 2.5 Immunofluorescence assay

Cells were fixed with 4% paraformaldehyde. The primary antibodies included OCT4 (1:200; Abcam, Cambridge, UK), SOX2 (1:400; CST, Danvers, MA, USA), SSEA4 (1:200; CST), MBP (1:100; Santa Cruz Biotechnology, Santa Cruz, CA, USA), GFAP (1:100; CST), S100β (1:100; Abcam), Nestin (1:100; Abcam), Neurofilament H (1:100; CST), and βIII‐tubulin (1:100; CST), PDGFRα(1:100, Santa cruz), NG2 (1:100, Santa cruz), αB‐Crystalline (1:200, Abcam). Secondary antibodies 488/568 goat antimouse/rabbit IgG (Invitrogen) were used and diluted at 1:500. The cell nuclei were counterstained with Hoechst33342 (Invitrogen). The labeled cells were imaged with a confocal microscope (Olympus FluoView FV10i, Tokyo, Japan).

### 2.6 In vitro differentiation of iPSCs

Vanishing white matter disease and control iPSCs more than 10 passages were differentiated into NSCs and then sequentially differentiated into neural cells, including neurons, oligodendrocytes (OLs), and astrocytes. Markers in different differentiation stages were detected. The fluorescence density was quantitatively detected by the following method: 8‐10 fields were randomly selected, including at least five areas in each field, then the mean fluorescence density in each area was calculated.

#### 2.6.1 Differentiation into NSCs

Induced pluripotent stem cells colonies were digested with EDTA when the iPSCs reached 70%‐80% confluence and then cultured in an E8 medium for a day. For 2‐7 days, the cells were cultured in a neural induction medium (Stemcell), and NSCs were formed and cultured in an NSC medium (DMEM/F12: Neural basal medium = 1:1 supplemented with 1 × N2, 1 × B27, 20 ng/mL bFGF). Immunofluorescence assay was used to detect the markers Nestin and SOX2.

#### 2.6.2 Differentiation into neurons

Neural stem cells were digested into single cells and then plated onto a Matrigel‐coated culture dish and cultured in a neuronal medium (bFGF removed from the NSC medium). By 7 days, the markers βIII‐tubulin and Neurofilament H were detected.

#### 2.6.3 Differentiation into astrocyte lineages

Neural stem cells were dissociated into single cells using Accutase and cultured in an astrocyte differentiation medium (Stemcell), and the cells were passaged every 4 days, both Nestin and CD44, indicative of immature astrocytes, were detected by immunofluorescence assay. By 12 days, the cells were digested and cultured in an astrocyte maturation medium (Stemcell). After at least one passages, the markers of GFAP and S100β, which are indicative of mature astrocytes, were detected by immunofluorescence assay. The number of processes and the length of the longest process from the margin of the nuclei were measured by calculating 10 randomly selected fields (cell number >5).

#### 2.6.4 Differentiation into OL lineages

Neural stem cells were dissociated into single cells and transferred to a wall of a six‐well plate coated with Matrigel at a density of 5 × 10^4^/cm^2^. The next day, the culture medium was changed to OL precursor cell (OPC) medium (DMEM/F12: neural basal medium = 1:1 supplemented with 1 × N2, 1 × B27, 0.4 μmol/L SAG, 20 ng/mL bFGF, and 20 ng/mL PDGF‐AA). After 7 days, the markers of NG2 and PDGFRα, which are indicative of OPCs, were detected by immunofluorescence assay. To differentiate the OPCs into OLs, the OPCs were dissociated into single cells and plate‐cultured in a wall of a six‐well plate coated with Matrigel at a density of 5 × 10^4^/cm^2^. The next day, the culture medium was changed to an OL medium (DMEM/F12: neural basal medium = 1:1 supplemented with 1 × N2, 1 × B27, 0.4 μmol/L SAG, 30 ng/mL T3, 10 ng/mL NT3, 10 μmol/L cAMP, and 100 ng/mL IGF‐1).^13^ MBP, indicative of mature OLs, was detected by immunofluorescence assay after the cells were cultured into OLs for 4 days.

### 2.7 Apoptosis analysis

The cells were digested with trypsin without EDTA. According to the instructions of the apoptosis kit (Roche, Basel, Switzerland), 100 μL of binding buffer solution was added to each sample, mixed with 2 μL of Annexin V and 2 μL of PI, and light reaction was avoided for 15 minutes at room temperature. Apoptosis was detected by flow cytometry (BD, San Jose, CA, USA) within 1 hour and analyzed with FlowJo software (TreeStar, Ashland, OR, USA).

### 2.8 Western blot analysis

For detecting total GFAP protein expression in astrocytes, total protein was extracted by RIPA buffer. Antibody to GFAP (mouse monoclone, 1:500, CST) was used in routine Western blot analysis. The expression of GAPDH (mouse monoclone, 1:1000) was used as a loading control.

### 2.9 Statistical analysis

Statistical analysis was performed with SPSS 20.0 (Chicago, IL, USA). The ANOVA analysis was adopted to determine the statistical significance in the following assays: the number of processes and the length of the longest astrocytic process, apoptosis detection, fluorescence density, *P* < 0.05 was considered statistically significant.

### 2.10 Ethical approval and consent forms

This study was approved by the clinical research ethics committee of the Peking University First Hospital, and informed consent forms were signed by the parents of the patients.

## 3 RESULTS

### 3.1 Phenotype and genotype of the two patients

**Figure 1:**
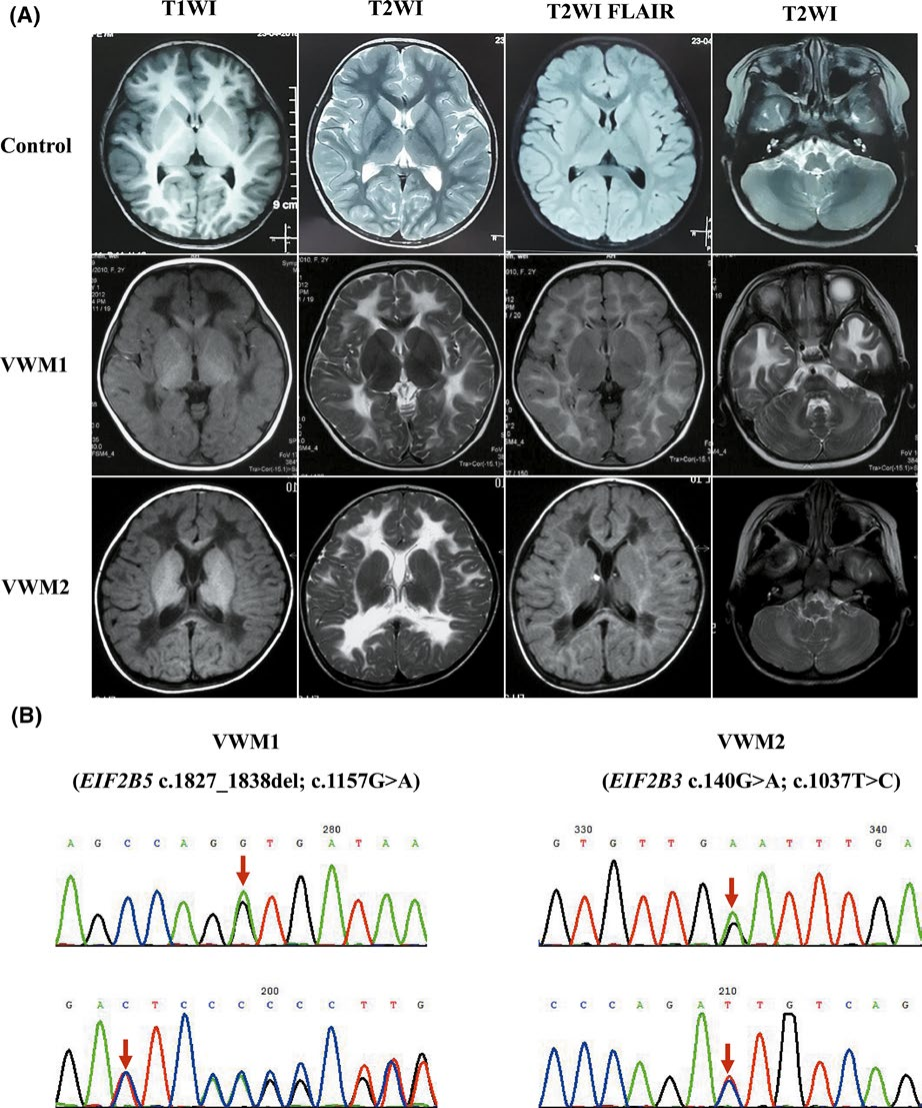
Features of the two VWM patients. a, Brain MRI of patients VWM1 and VWM2 and an age‐matched control, performed at the age of 4, 3, and 4, respectively: A, E, and I (T1WI); B, D, F, K, J, and L (T2WI); C, G, and K (T2 FLAIR). Brain MRI of both VWM patients showed symmetric abnormal signals on T1WI, T2WI, and T2 FLAIR in the white matter (WM), which was partially rarefied. b, Direct sequencing analysis of the genomic DNA from the two VWM patients’ iPSCs showed VWM1: *EIF2B5* c.1827_1838del (p. Ser610_Asp613del), c.1157G>A (p.Gly386Val); VWM2: *EIF2B3* c.140G>A (p. Gly47Glu), c.1037T>C (p. Ile346Thr)

Two VWM iPSC models (VWM1‐iPSCs and VWM2‐iPSCs), which are *EIF2B5* and *EIF2B3* compound heterozygous mutations, respectively, were established using a nonintegrating episomal vector system. Both patients were early childhood onset VWM, the developmental milestone before disease onset was normal. The first patient (VWM1) was a male, whose initial symptom was motor deterioration triggered by head trauma at 4 years old. At the last follow‐up in 2018, he was 16 years old and was bedridden. The second patient (VWM2) was a female, who was characterized by motor regression at the age of 3 and died at 12 years old. The brain MRI showed typical features of rarefaction of cerebral white matter (Figure 1a). The genotype of VWM1 was *EIF2B5*: c.1827_1838del (p. Ser610_Asp613del), c.1157G>A (P. Gly386Val); whereas the genotype of VWM2 was *EIF2B3*: c.140G>A (p. Gly47Glu), c.1037T>C (p. Ile346Thr) (Figure 1b).

### 3.2 Establishment and characterization of the VWM HDFs‐derived iPSCs

**Figure 2:**
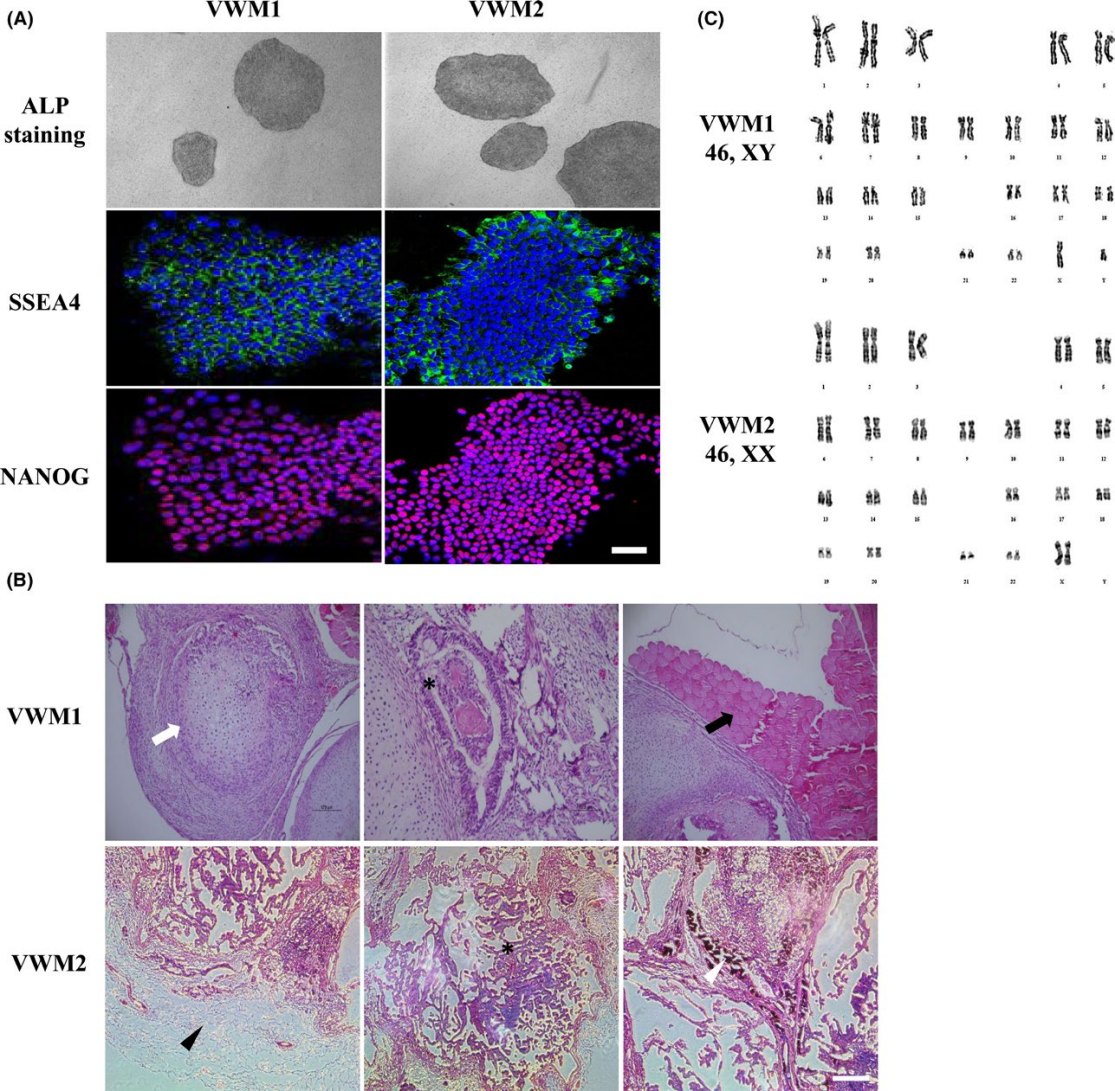
Characterization of VWM iPSCs. a, Positive alkaline phosphatase staining showed typical morphology of iPSC clones (top) and immunochemical analysis of pluripotent markers, SSEA4, and NANOG (bottom). b, Representative hematoxylin and eosin staining of teratomas derived from the established VWM iPSC clones. The teratomas were formed via the subcutaneous injection of undifferentiated iPSCs into the posterior leg of NOD/SCID mice. VWM1: Open arrow, cartilage; asterisks, respiratory epithelia; arrow, muscle. VWM2: Arrowhead, adipocyte; asterisks, gut‐like epithelia; open arrowhead, pigmented epithelia. The scale bar represents 200 μm. c, Karyotype analysis showed normal karyotypes of VWM iPSCs (more than 10 passages), 46, XY and 46, XX, respectively

Forearm dermal tissue was obtained from the two VWM patients at the age of 10 and 11, respectively. To generate integration‐free iPSCs, the Yamanaka episomal plasmids were electrotransfected into the HDFs of the two VWM patients using an Amaxa Nucleofector system. 25‐30 days after electrotransfection, clones were picked and propagated under feeder‐free conditions. iPSCs clones exhibited compact and flat appearance, similar to ESCs. In addition, iPSCs stained positive for alkaline phosphatase (ALP) staining and expressed typical pluripotent markers, SSEA4, and NANOG (Figure 2a). Furthermore, in vivo teratoma formation assay was performed in NOD SCID mice. After 2 months, teratomas were formed, and histological examination showed that the teratomas were composed of cells characterized by three germ layers (Figure 2b). Karyotype analysis showed that VWM1 and VWM2 iPSCs maintained normal karyotypes after 10 passages, namely, 46, XY and 46, XX, respectively (Figure 2c). Sanger sequencing confirmed that the VWM1 and VWM2 iPSCs carried the same mutations as the fibroblasts.

### 3.3 VWM iPSCs differentiated into NSCs in vitro

**Figure 3:**
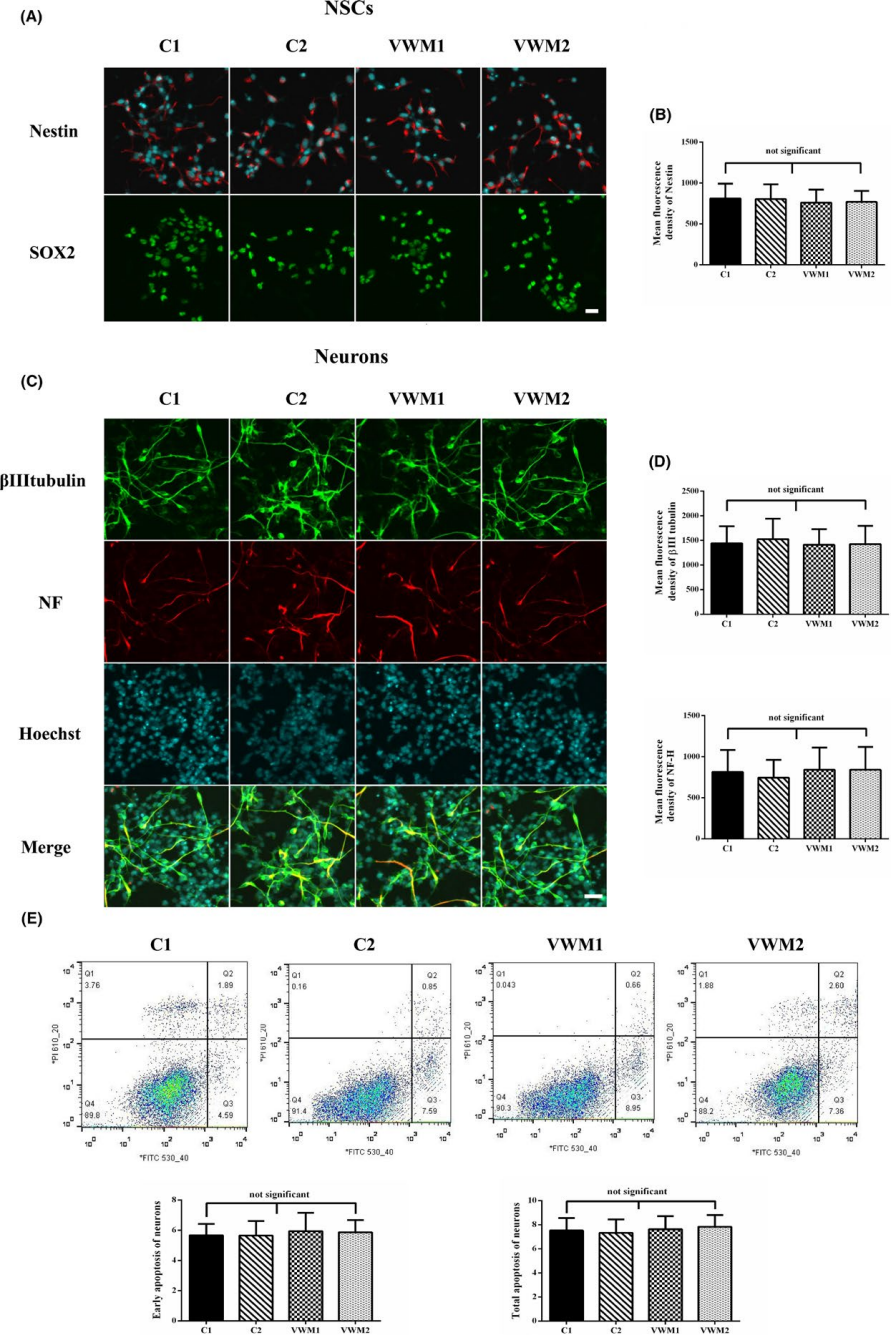
Differentiation of iPSC‐derived NSCs into neurons. a, Immunochemical analysis of iPSC‐derived NSCs, NSCs were positive for Nestin and SOX2. b, Mean fluorescence densities of Nestin of NSCs, no significant difference exists, *P* > 0.05. c, Representative image of the immunochemistry of the NSC‐derived neurons. The neurons were positive for βIII‐tubulin (green color) and Neurofilament H (red color) after 7 d of NSC differentiation. The scale bar represents 30 μm. d, Mean fluorescence densities of βIII‐tubulin and Neurofilament H of neurons; no significant difference exists, *P* > 0.05. e, Quantification of the early and total apoptosis of the neurons showed no significant differences between the Control, VWM1, and VWM2 neurons (*P* > 0.05, biological replicates, n = 3)

Vanishing white matter disease iPSCs and two control iPSCs lines (C1 and C2) were induced to differentiate into NSCs by using neural induction medium. After two passages, both control and VWM iPSCs expressed Nestin and SOX2. Nestin was localized in the cytoplasm, whereas SOX2 was in the nuclei (Figure 3a). In addition, the mean fluorescence densities of Nestin in the C1, C2, VWM1, and VWM2 NSCs were 813.7, 805.5, 760.4, and 768.9, respectively, *P* > 0.05 (Figure 3b).

### 3.4 VWM iPSC‐derived NSCs differentiated into neurons in vitro

The control and VWM NSCs after two passages were induced to differentiate into neurons. On day 7, both βIII‐tubulin and Neurofilament H were positive in the control, VWM1, and VWM2 NSCs‐derived neurons, and were localized in the cytoplasm (Figure 3c). In addition, the mean fluorescence densities of βIII‐tubulin in the C1, C2, VWM1, and VWM2 NSCs were 1375, 1388, 1393, and 1399, *P* > 0.05, whereas those of Neurofilament H were 814.8, 744.5, 841.3 and 831.5, respectively, *P* > 0.05 (Figure 3d).

The apoptosis of neurons was further analyzed by AnnexinV/PI. The results revealed that the early apoptosis rates of the C1, C2, VWM1, and VWM2 neurons were 4.59%, 7.59%, 8.95%, and 7.36%, respectively, and their total apoptosis rates were 6.48%, 8.44%, 9.61%, and 9.96%, respectively. There is no significant difference in either early and total apoptosis rates between the controls and VWM neurons, *P* > 0.05 (Figure 3e).

### 3.5 VWM iPSC‐derived NSCs differentiated into OLs in vitro

**Figure 4:**
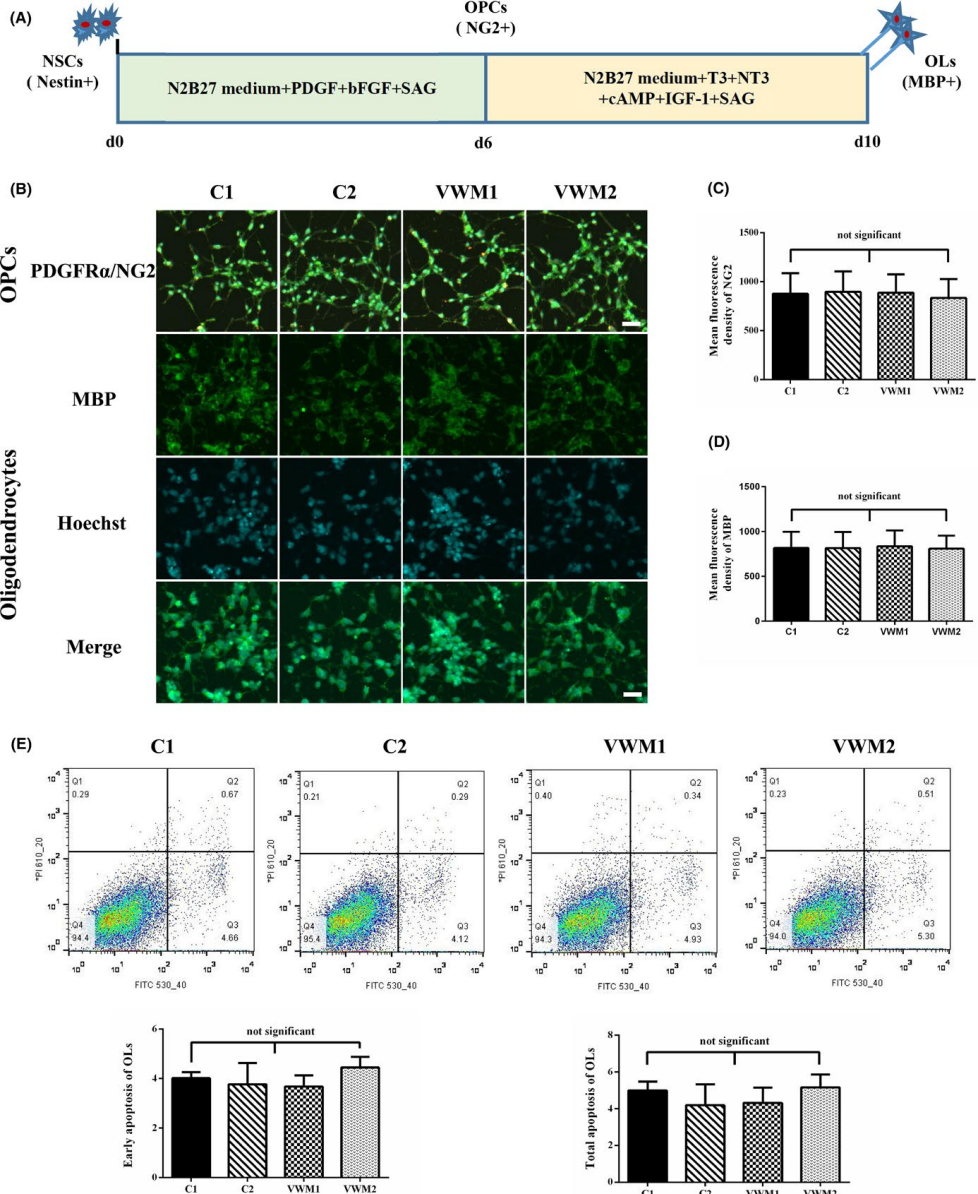
Differentiation of iPSC‐derived NSCs into oligodendrocytes. a, Schematic presentation of the protocol for OLs differentiation from NSCs. b, Immunochemical analysis of iPSC‐derived OPCs and OLs. Both control and VWM OPCs were positive for NG2 (green color) and PDGFRα (red color) after 6 d of NSCs differentiation. And both control and VWM OLs were positive for MBP after 4 d of OPCs differentiation. The scale bar represents 30 μm. c, d, Mean fluorescence densities of NG2 and MBP respectively; no significant difference exists, *P* > 0.05. e, Apoptosis detection of OLs via Annexin V/PI staining. Quantification of the early and total apoptosis of the OLs showed no significant differences between the controls, VWM1, and VWM2 OLs (*P* > 0.05, biological replicates, n = 3)

The NSCs were differentiated into OLs by the standard protocol (Figure 4a). After the NSCs were cultured in the OPC medium for 7 days, OPCs derived from both control and VWM NSCs expressed PDGFRα and NG2 (Figure 4b). Both PDGFRα and NG2 were localized in the cytoplasm, the mean fluorescence densities of NG2 in C1, C2, VWM1, and VWM2 OPCs were 817.0, 815.3, 835.6, and 809.8 respectively (*P* > 0.05) (Figure 4c).

The OPCs were further differentiated into OLs. After 4 days, both the control and VWM OPCs expressed MBP, which was localized in the membrane (Figure 4b). The mean fluorescence densities of MBP in the C1, C2, VWM1, and VWM2 OLs were 817.0, 815.3, 835.5, and 809.7, respectively (*P* > 0.05) (Figure 4d).

The apoptosis of OLs was analyzed by AnnexinV/PI, which revealed that the early apoptosis rates of the C1, C2, VWM1, and VWM2 OLs were 4.66%, 4.12%, 4.93%, and 5.30%, respectively, and their total apoptosis rates were 5.33%, 4.41%, 5.27%, and 5.81%, respectively. No significant difference exists in early and total apoptosis rates between the controls, VWM1, and VWM2 OLs, *P* > 0.05 (Figure 4e).

### 3.6 VWM iPSC‐derived astrocytes exhibited abnormal morphology and increased apoptosis

#### 3.6.1 No difference found in the timing of markers in different stages of differentiation between control and VWM astrocytes

**Figure 5:**
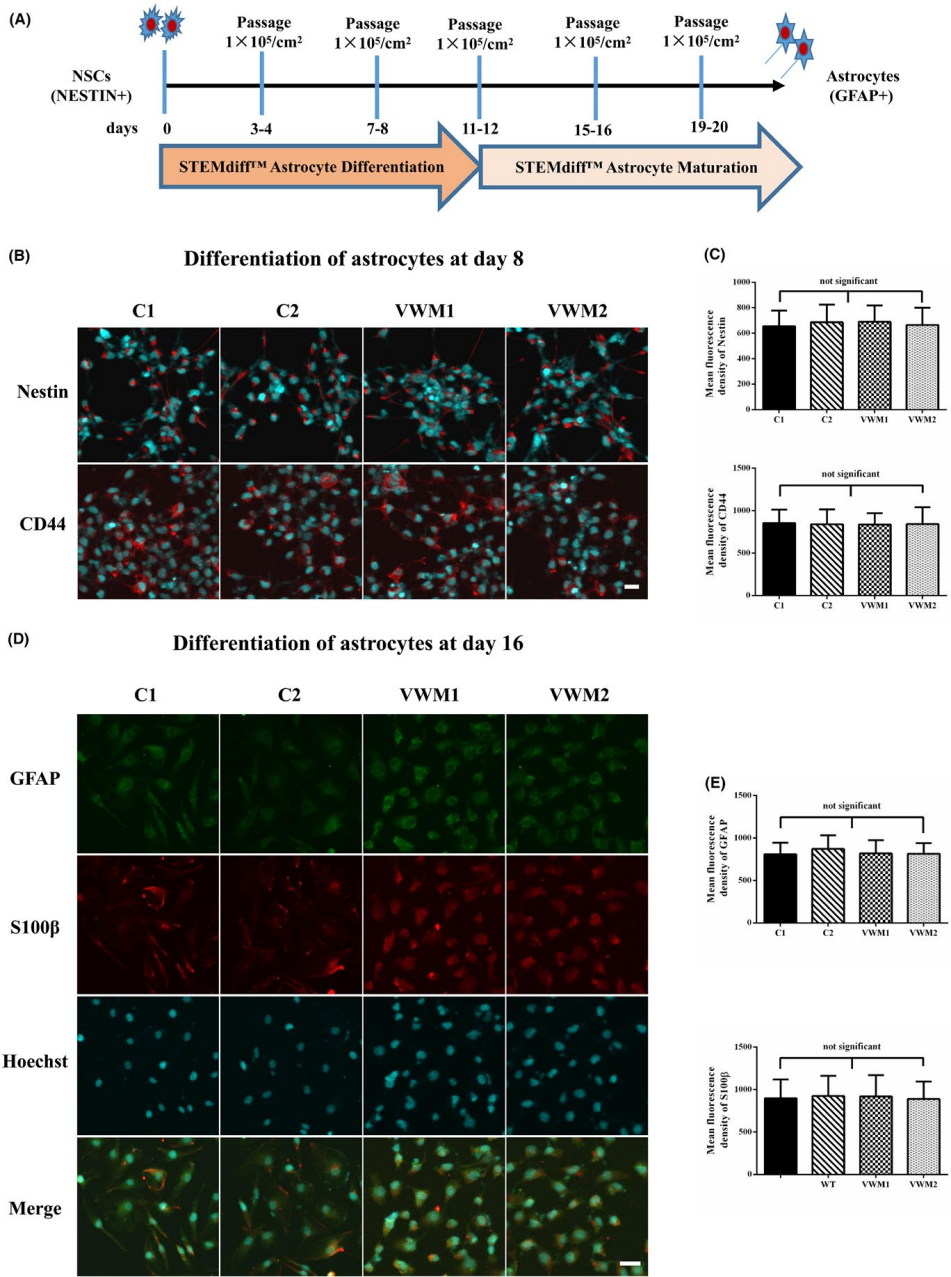
Differentiation of iPSC‐derived NSCs into astrocytes. a, Schematic presentation of the protocol for astrocytes differentiation from NSCs. b, Cells were positive for Nestin and CD44 after 8 d of NSC differentiation into astrocytes. The scale bar represents 30 μm. c, Mean fluorescence densities of Nestin and CD44, respectively; no significant difference exists, *P* > 0.05. d, Both control and VWM Astrocytes were positive for GFAP (green color) and S100β (red color) after 16 d of NSCs differentiation into astrocytes. The scale bar represents 30 μm. e, Mean fluorescence densities of GFAP and S100β, respectively; no significant difference exists, *P* > 0.05

The NSCs were differentiated into astrocytes according to the standard protocol (Figure 5a). On day 8, both control and VWM cells expressed Nestin and CD44, indicative of astrocyte precursor cells (Figure 5b). Nestin was localized in the cytoplasm, and the mean fluorescence densities of Nestin in the C1, C2, VWM1, and VWM2 cells were 655.4, 686.3, 689.6 and 665.1, respectively (*P* > 0.05). CD44 was localized in the membrane, and the mean fluorescence densities of CD44 in the C1, C2, VWM1, and VWM2 cells were 856.1, 840.9, 838.1, and 843.4, respectively (*P* > 0.05) (Figure 5c). On day 16, both control and VWM cells expressed mature astrocytic markers GFAP and S100β (Figure 5d), both GFAP and S100β were localized in the cytoplasm, and the mean fluorescence densities of GFAP in the C1, C2, VWM1, and VWM2 cells were 808.7, 871.7, 819.6, and 815.9 (*P* > 0.05), and those of S100β were 897.9, 923.8, 918.9, and 889.6 (*P* > 0.05) (Figure 5e).

#### 3.6.2 VWM iPSC‐derived astrocytes were dysmorphic

**Figure 6:**
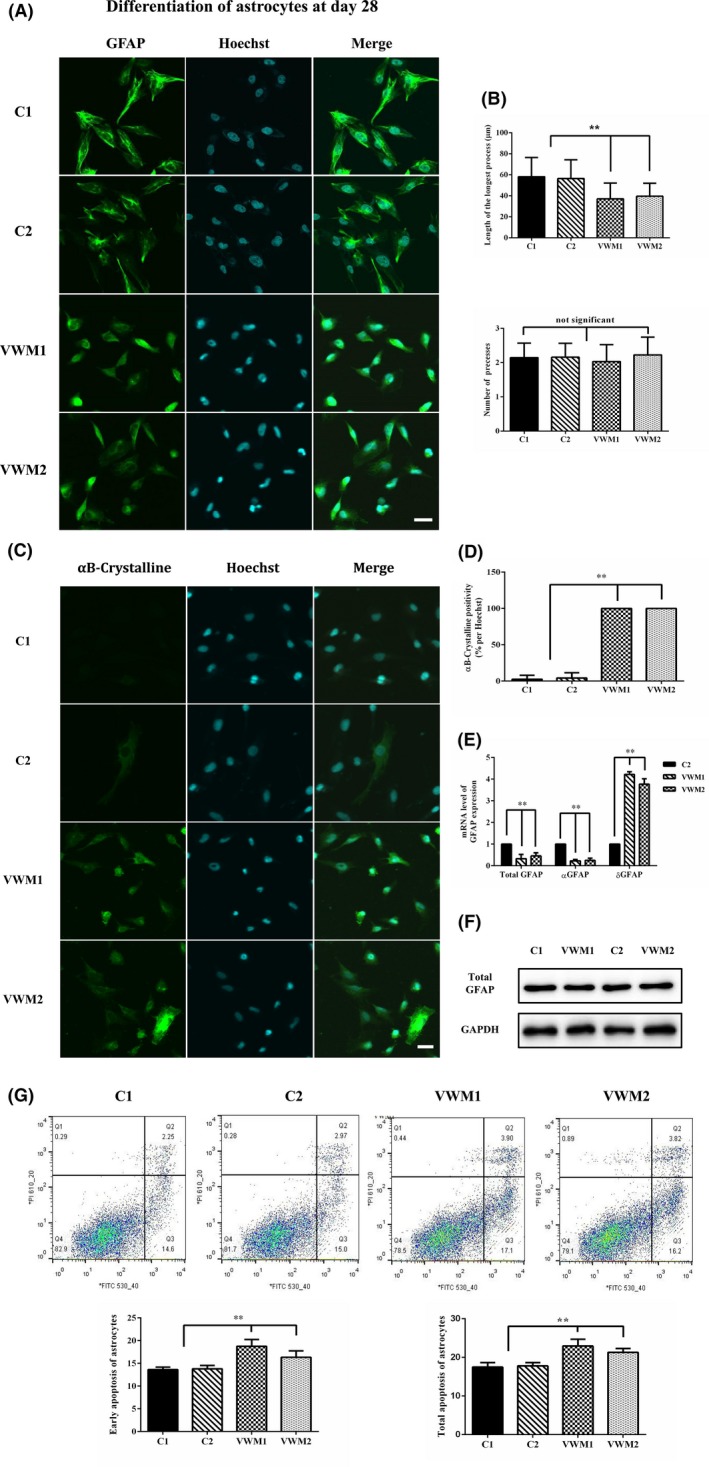
Involvement of VWM iPSC‐derived astrocytes. a, Representative image of the immunochemistry of control and VWM astrocytes. The mature astrocytes were positive for GFAP after 28 d of NSCs differentiation into astrocytes. The scale bar represents 30 μm. b, Calculated length of the longest astrocytic process and number of processes of the astrocytes (10 fields were randomly selected, with at least 8 cells in each field). c, Immunochemical analysis of the expression of αB‐Crystalline in control and VWM astrocytes. The scale bar represents 30 μm. d, Calculated positivity of αB‐Crystalline‐positive (αB‐Crystalline/Hoechst) astrocytes (**, *P* < 0.0001, 10 fields were randomly picked, with at least 8 cells in each field). e, Real‐time quantitative PCR analysis for GFAP (total, αGFAP and δ‐GFAP) expression in Control and VWM astrocytes (**, *P* < 0.01 in the three groups, biological replicates, n = 3). f, The total GFAP expression in Control and VWM astrocytes detected by Western blot exhibited no significant difference. g, Apoptosis detection in the astrocytes via Annexin V/PI staining and quantification of total and early apoptosis of astrocytes (** represents *P* < 0.01, * represents *P* < 0.05, biological replicates, n = 3)

On day 28 of differentiation, the VWM1 and VWM2 astrocytes were significantly dysmorphic, manifested as relatively shorter processes (Figure 6a). The number of processes and the length of the longest astrocytic process were analyzed using phase contrast microscopy. The mean length of the longest process of the control astrocytes was 58.1 and 56.4 μm, whereas those of the VWM1 and VWM2 astrocytes were 37.1 and 39.5 μm, respectively (*P* < 0.0001). The numbers of processes between the control and VWM astrocytes did not demonstrate significant difference, *P* > 0.1 (Figure 6b).

#### 3.6.3 Overexpression of αB‐Crystalline in VWM astrocytes

Increased expression of protein chaperon αB‐crystalline was determined in VWM astrocytes (Figure 6c). αB‐Crystalline positivity (αB‐Crystalline/Hoechst) were further analyzed, and 100% of VWM1 and VWM2 astrocytes expressed αB‐Crystalline compared with less than 5% of control astrocytes, *P* < 0.0001 (Figure 6d).

#### 3.6.4 Increased expression of δ‐GFAP in VWM astrocytes

The GFAP expression in astrocytes was detected by RT‐qPCR. The expression levels of total GFAP and α‐GFAP in the VWM astrocytes were lower than those in the control astrocytes. By contrast, the expression levels of δ‐GFAP in the VWM astrocytes were significantly higher than those in the control astrocytes, *P* < 0.01 (Figure 6e). The total GFAP expression in control and VWM astrocytes detected by Western blot exhibited no significant difference (Figure 6f).

#### 3.6.5 Increased apoptosis in VWM astrocytes

The apoptosis of astrocytes was detected on day 28 of differentiation. The early apoptosis rates of the C1, C2, VWM1, and VWM2 astrocytes were 14.6%, 15.0%, 17.1%, and 16.2%, respectively, and their total apoptosis rates were 16.8%, 17.9%, 21.0%, and 20.0%, respectively. Both the early and total apoptosis rates of the VWM astrocytes were higher than those of the control astrocytes, *P* < 0.05 (Figure 6g).

Overall, compared with the control astrocytes, the VWM astrocytes exhibited abnormal morphology, expressed abnormal antigenic phenotypes, and manifested increased total and early apoptosis, suggesting that VWM astrocytes were dysfunctional and involved in the pathogenesis of VWM.

## 4 DISCUSSIONS

### 4.1 Advantages and disadvantages of disease models of VWM

The exact pathogenesis of VWM remains unknown. Current studies of VWM mainly concentrated in the following aspects: the overactivation of unfolded protein reaction (UPR), mitochondrial dysfunction, and glial maturation dysfunction.^9^, ^14^, ^15^, ^16^, ^17^ Previous studies mainly used patients’ postmortem brain tissue, animal models, or cell transfection. The postmortem brain tissue VWM patients can reflect the pathological features at the tissue and cellular levels. However, the brain tissue is usually difficult to obtain, and can only reflect the terminal state rather than the dynamic changes in the disease. Animal models are more readily available and can be used for multilevel studies, but the phenotypes of mice and VWM patients are not completely parallel.^18^, ^19^ Currently, in vitro virus‐mediated cell transfection is widely used; however, viral genome may be randomly integrated into the host cell genome. In addition, the overexpression of target gene could not reflect the physiological situation or maintain stable transfection.^22^, ^23^ In our study, VWM disease‐specific iPSC models were established for the first time, however, several main disadvantages of iPS models such as new variants, epigenetic modification, and tumor formation remain unsolved, which prevent utilization of iPSCs in clinical application like in vivo transplantation.

### 4.2 VWM glial cells are selectively involved, but neurons are spared

The typical brain MRI of VWM patients shows that the cerebral white matter is diffusely rarefied, whereas the cortex is relatively well preserved.^7^, ^24^, ^25^ In addition, the histomorphology of the postmortem brain tissue of VWM patients and mouse models suggested that myelin is lost, and white matter is liquefied or vacuolated, whereas the gray matter looks normal. Histopathological evaluation indicated that astrocytes show abnormal morphology and decreased reactive gliosis, increased foaming OLs and apoptosis, whereas neurons are relatively normal.^27^, ^28^ In the current study, VWM iPSC‐derived NSCs could normally differentiate into neurons, further supporting that neurons are spared in VWM.

### 4.3 Astrocytes may play a central role in the pathogenesis of VWM

In the central nervous system (CNS), astrocytes account for the largest number of cells and play a central role in the maintenance of homeostasis, the response to injury, and the pathogenesis of disease. Astrocytes participate in a series of complex pathological processes, such as reactive gliosis, antioxidant, and immune regulation.^30^, ^31^

Several studies have suggested that astrocytes are central in the pathogenesis of VWM. Dietrich et al^33^ cultured astrocytes and OLs in vitro from the brain tissue of a VWM patient with *EIF2B5* mutation; they found that few GFAP+astrocytes were present and astrocytic induction was severely compromised, whereas normal OLs can be cultured. Detailed VWM pathological examination has revealed meager reactive astrogliosis, dysmorphic astrocytes, and increased expression of delta isoform GFAP (δ‐GFAP) and heat shock protein αB‐crystalline.^34^ Although in vitro evidence has confirmed that astrocytes are primarily impaired, the postmortem brain tissue and animal models of VWM have suggested that OLs are also involved, showing that the OLs are foamy and the number of myelin‐forming OLs were decreased.^3^, ^27^, ^34^, ^35^ In addition, Van Haren et al^37^ found that OLs increased in number but also demonstrated limited proliferation and increased apoptosis in VWM. We also found in our previous studies that OLs transfected with mutant eIF2B showed ERS intolerance, overactivation of UPR and decreased autophagy.^38^, ^39^

In our study, we found that VWM iPSC‐derived NSCs can normally differentiate into OPCs, and OLs in vitro. Whereas, VWM iPSC‐derived astrocytes were dysmorphic, expressed a significant increased δ‐GFAP and αB‐Crystalline, and showed increased early and total apoptosis as well, which indicating the astrocytic dysfunction. Dysmorphic astrocytes overexpressed δ‐GFAP, suggesting that the intermediate fiber network of VWM astrocytes was affected, resulting in abnormal morphology and meager astrogliosis.^3^, ^40^ Previous studies showed that astrocytes can influence OPC survival, differentiation, and maturation.^41^, ^42^ Typical neuropathological findings showed that axons are lost in cavitated white matter and remaining axons are abnormally thin. Klok, et al^45^ proposed that axons are initially normal and atrophy later in VWM, and astrocytes are central in this process. Bugiani et al^34^, ^46^ found myelin vacuolation and increased density of OPCs with normal proliferation in the brain tissue of VWM patients, whereas VWM astrocytes inhibited the differentiation of OPCs into mature myelin‐forming OLs. Dooves et al^47^ found in their coculture experiments that VWM astrocytes could inhibit the maturation of wild‐type OLs. Therefore, mutant astrocytes may play a major role in the vanishing of white matter by disturbance of function and survival of OLs. In the newly proposed classification system of leukodystrophy, VWM was classified as an astrocytic disease.^48^

In addition to the pathological changes in astrocytes in cerebral white matter, astrocytes in other regions can also be involved. Both in VWM patients and mouse model of VWM, Bergmann glia in the cerebellum and Müller cells in the retina are also affected. The Bergmann glia in the cerebellum shows mislocalization to the molecular layer, and the involvement of Müller glia was manifested as retinal dysplasia. Retrospective evaluation of patients’ electroretinographic data confirmed that retinopathy is also a sign of VWM patients.^40^, ^47^ Moreover, Leferink et al^49^ also identified astrocytic abnormalities in the spinal cord of a mouse model for VWM and the postmortem tissue of two VWM patients.

### 4.4 Limitations

There must be some differences between iPSC differentiation in vitro and neural differentiation in vivo. Moreover, although iPSCs carry VWM mutations, new variants, and epigenetic changes may occur during the processes of iPSCs reprogramming and differentiation.

## 5 CONCLUSIONS

In this study, two VWM iPSC models were established using a virus‐free nonintegrating episomal vector system for the first time. The results suggested that there was no difference in the in vitro differentiation of control and VWM iPSCs into NSCs, neurons, and OLs. Whereas, VWM astrocytes exhibited abnormal morphology, increased expression of δ‐GFAP and αB‐Crystalline, and increased early and total apoptosis, further supporting that astrocytes may play a central role in the pathogenesis of VWM. In addition, the established VWM‐specific iPSCs models provide a platform for further study on the pathogenesis and future drug screening.

## CONFLICT OF INTEREST

The authors declare no conflict of interest.
